# Evaluation of prescribing adherence to guideline-directed medical therapy in patients with chronic heart failure: a retrospective study at the National Heart Centre in Oman

**DOI:** 10.1186/s12872-025-05232-3

**Published:** 2025-10-24

**Authors:** Manal Abdullah Albalushi, Leila Neshat-Mokadem, Ghalib Al-Mawaali

**Affiliations:** 1 Department of Pharmaceutical Care, The Royal Hospital, Muscat, Oman; 2 School of Nursing and Health Sciences, Robert Gordon University, Aberdeen, Scotland, UK

**Keywords:** Guideline-directed medical therapy, Heart failure, HFrEF, Prescribing adherence, Oman, Target dose

## Abstract

**Background:**

Guideline-directed medical therapy (GDMT) improves survival and quality of life in patients with heart failure with reduced ejection fraction (HFrEF). However, real-world adherence to these guidelines remains suboptimal. This study aimed to evaluate prescribing adherence to GDMT in HFrEF patients using a global guideline adherence scoring tool.

**Methods:**

This retrospective study included adult patients with HFrEF (left ventricular ejection fraction ≤ 40%) attending the Heart Failure Clinic at the National Heart Centre in Oman between January and June 2022. GDMT adherence was assessed based on 2021 European Society of Cardiology guidelines. Adherence levels were categorized as good (all indicated GDMT at ≥ 50% of target dose), moderate (more than half at ≥ 50%), or poor (less than half or < 50%). Descriptive statistics and chi-square tests were used to identify associations.

**Results:**

Of the 259 patients included (mean age 57 ± 13.6 years, 70% male), 71% had good adherence, 22% moderate, and 7% poor. Target dose attainment varied by drug class: 78% for beta-blockers, 63% for ACEI/ARB/ARNI, 96% for MRAs, and 100% for ivabradine. Suboptimal GDMT (< 50% target dose) was significantly associated with hypertension (*P* = 0.004), dilated cardiomyopathy (*P* = 0.015), chronic kidney disease (*P* = 0.001), and older age (*P* = 0.004).

**Conclusion:**

Prescribing adherence to GDMT in Oman is consistent with international data. Suboptimal titration was significantly linked to older age and comorbidities, suggesting that perceived frailty and clinical complexity may influence physician prescribing patterns. Efforts to improve individualized, patient-centered GDMT titration are warranted.

## Background

Heart failure (HF) is a growing global health challenge, affecting over 64 million individuals worldwide, and contributing significantly to hospitalizations, healthcare costs, and mortality rates [1, 2]. Among its classifications, heart failure with reduced ejection fraction (HFrEF) defined as a left ventricular ejection fraction (LVEF) of ≤ 40% is a well-characterized subtype with established therapeutic options [3]. Guideline-directed medical therapy (GDMT), comprising β-blockers (BBs), angiotensin-converting enzyme inhibitors (ACEIs) or angiotensin receptor blockers (ARBs), angiotensin receptor–neprilysin inhibitors (ARNIs), mineralocorticoid receptor antagonists (MRAs), and sodium-glucose cotransporter-2 inhibitors (SGLT2is), has been shown to reduce morbidity and mortality when prescribed appropriately and titrated to target doses [4–8].

Despite the availability of robust international guidelines and proven benefits of GDMT, several large-scale registries have shown that many patients with HFrEF receive suboptimal treatment either due to the omission of key medication classes or failure to up-titrate to evidence-based target doses [9–13]. Notably, patients treated with less than 50% of the recommended doses may not experience the full survival benefit seen in clinical trials [9].

This treatment gap is more pronounced in real-world settings, particularly in low- and middle-income countries, where clinical inertia, limited awareness of guidelines, concerns about tolerability, and health system barriers all contribute to underutilization of GDMT [14–17]. In the Middle East and specifically in Oman, data on GDMT prescribing patterns are limited. Although regional studies such as the Gulf DYSPNEA registry have highlighted general prescribing trends [10], there remains a paucity of focused, patient-level evaluations of adherence to GDMT in specialized heart failure clinics in Oman. Without such data, it is difficult to tailor local quality improvement efforts or understand context-specific barriers to optimal HF management.

Treatment adherence is a critical determinant of outcomes in cardiovascular disease, and simplifying dosing frequency is one practical lever to improve adherence. Real-world data show high adherence to lipid-lowering biologics administered infrequently (e.g., monthly PCSK9 monoclonal antibodies), supporting the concept that less-frequent dosing can facilitate persistence with therapy [18]. More broadly, contemporary HF registries underscore that rapid implementation and consistent up-titration of the four foundational HFrEF drug classes are often not achieved in routine care, highlighting the need for strategies including simplified regimens and reduced dosing frequency where feasible to close adherence gaps [19]. While most GDMT agents in HFrEF are oral daily therapies, these observations from cardiovascular pharmacotherapy more generally (e.g., monthly/biannual injectable lipid-lowering agents) reinforce the principle that minimizing dosing burden may be an effective adherence strategy to consider alongside fixed-dose combinations, pharmacy synchronization, and team-based follow-up within HF programs.

The Global Guideline Adherence Score, developed by Komajda et al., offers a practical framework to evaluate real-world prescribing patterns and has been used in several countries [14]. However, to our knowledge, this tool has not been applied in the Omani healthcare setting or the broader Gulf region. Therefore, the present study aimed to evaluate prescribing adherence to GDMT among HFrEF patients attending a specialized heart failure clinic at the National Heart Centre in Muscat, Oman, using the Global Guideline Adherence Score. Additionally, we assessed whether suboptimal adherence (< 50% of target dose) was associated with patient demographics and comorbid conditions, to better understand the local challenges to optimal HF management.

## Methods

### Study design and population

Patients with chronic heart failure (HF) (≥ 18 years of age) who visited the HF Clinic at the National Heart Centre (NHC) in Muscat, Oman, between January 1, 2022, and June 30, 2022, were assessed for eligibility. All patients with LVEF ≤ 40% measured on the most recent echocardiogram (≤ 2 years) were enrolled. If a patient had multiple ejection fraction (EF) measurements, the most recent one was used. Eligible patients had a documented diagnosis of HF from a hospital admission at least 3 months before enrolment and an echocardiogram confirming the diagnosis. The study uses a convenience sample, a non-probability sampling method, since no previous data exist on local HFrEF prevalence.

### Setting

NHC provides comprehensive care for cardiovascular conditions and heart diseases in Muscat, Oman. It is considered a referral tertiary care health institution that receives patients from all regional primary and secondary health care institutions (hospitals, polyclinics and health centres) in Oman. At NHC, HF patients are treated and followed up by cardiologists with sub-speciality in HF management with work experience ranging from 5 to 10 years.

### Data collection and generation

Demographic and clinical characteristics for this study were collected from the NHC’s data registry, Al-Shifa 3Plus (the healthcare information system established by the Ministry of Health, Oman). Additionally, GDMT (ACEI/ARB/ARNI, BB, MRA, ivabradine) medications and their corresponding doses were also collected from patients’ electronic records. The optimum target doses for carvedilol, bisoprolol, lisinopril, valsartan, the sacubitril/valsartan combination, ivabradine, spironolactone, and eplerenone were identified according to the 2021 European Society of Cardiology (ESC) HF guidelines which was the most update guidelines for HF at the time study conducted and utilized by HF cardiologists at NHC. The target doses used for classification were as follows:

- Carvedilol: 25 mg twice daily (50 mg twice daily for patients > 85 kg)
- Bisoprolol: 10 mg once daily
- Lisinopril: 20–35 mg once daily
- Valsartan: 160 mg twice daily
- Sacubitril/valsartan: 97/103 mg twice daily
- Ivabradine: 7.5 mg twice daily
- Spironolactone: 25–50 mg once daily
- Eplerenone: 50 mg once daily

Dose attainment was classified as follows:

- Target dose attainment: receiving ≥ 50% of the guideline-recommended dose (this category also included patients who achieved 100% of the target dose).
- Full target dose attainment: receiving exactly 100% of the guideline-recommended dose.

Additionally, other medications for co-morbid conditions were also collected. The adherence score was calculated for each patient as follows: 1 point for each prescription of an ACEI/ARB/ARNI, BB, MRA, ivabradine (if indicated), and 0 points for no prescriptions. In the case of contraindications or intolerance, 1 point was given. Thus, for eligible patients,

1. Use of all indicated medications in doses ≥ 50% of the target dose is considered good adherence,
2. Use of more than half of the medications in doses ≥ 50% of the target dose, moderate adherence, and
3. Use of less than half the recommended medications and/or in doses < 50% of target dose, poor adherence.

### Statistical analysis

Continuous variables normally distributed were reported as mean ± standard deviation while categorical variables were reported as numbers and percentages. Chi-square test or Fisher’s exact were used to assess the association between categorical variables as appropriate. Statistical significance was defined as a *p*-value < 0.05. The Statistical Package for the Social Sciences (SPSS, version 28; IBM Corporation) was used to perform the statistical analyses.

### Outcome measures

The primary outcome measures of interest were the assessment of the use and corresponding dosages of GDMT in patients with HFrEF in Oman population using the Global guideline adherence score. Secondary outcome measures included determining the association between patients’ demographic characteristics, such as age, comorbidities, and gender, and the sub-optimisation of GDMT (< 50%) among the Omani HFrEF population.

## Results

A total of 259 patients with HFrEF met the inclusion criteria and were enrolled in the study. The mean left ventricular ejection fraction (LVEF) was 28 ± 6.9%. The average age of the cohort was 57 ± 13.6 years, and the majority were male (70%; *n* = 180). Current smoking was reported in 4.2% (*n* = 11) of patients.

Comorbidities were common among the study population: hypertension (HTN) was present in 42.1% (*n* = 109), diabetes mellitus (DM) in 35.1% (*n* = 91), dilated cardiomyopathy (DCM) in 32.8% (*n* = 85), coronary artery disease (CAD) in 18.1% (*n* = 47), and chronic kidney disease (CKD) in 12% (*n* = 31). As shown in Table 1, most patients were prescribed diuretics (78.4%; *n* = 203), and a significant proportion were on statins (56.8%; *n* = 147) and antiplatelet agents (43.6%; *n* = 113). Digoxin use was infrequent, reported in only 2.7% (*n* = 7) of patients.

**Table 1:** Demographic and clinical characteristics of study population

Characteristics, *n* (%) unless specified otherwise	Patients *n* = 259
Male	180 (69.5%)
Female	79 (30.5%)
Age, mean ± standard deviation (SD), years	57 ±13.6
Smoking status	
None-smoker	13 (5%)
Smoker	11(4.2%)
Ex-smoker	26(10%)
New York Heart Association Class	
I	58 (22.4%)
II	81(31.3%)
III	21 (8.1%)
IV	2 (0.8%)
Heart Rate, mean ± SD, beats per minute	75.32 ±14.29
Blood Pressure (millimetres of mercury, mmHg)	
Systolic blood pressure, mean ± SD, mmHg	132.34 ±22.49
Diastolic blood pressure, mean ± SD, mmHg	76.92 ±15.6
Ejection Fraction	
Less than 35%	194 (75%)
35% and more	65 (25%)
Serum Creatinine, mean ± SD, micromoles/litre	95.44 ±28.49
Haemoglobin, mean ± SD, grams/decilitre	13.48 ±4.09
Time since first Heart Failure diagnosis, mean ± SD, years	4.45 ±3.33
Co-morbidities	
Asthma/Chronic obstruction pulmonary disease	12 (4.6%)
Chronic kidney disease	31(12%)
Hypertension	109(42.1%)
Diabetes Mellitus	91 (35.1%)
Anaemia	3 (1.2%)
Atrial Fibrillation	27 (10.4%)
Coronary artery disease	47 (18.1%)
Stroke/Transient ischemic attack	3 (1.2%)
Left bundle branch block	26 (10%)
Ischemic Cardiomyopathy	27 (10.4%)
Dilated Cardiomyopathy	85 (32.8%)
Others	218 (84.2%)
Pharmacological treatment and devices	
Amiodarone	14 (5.4%)
Antidiabetics	83 (32%)
Anticoagulants	53 (20.5%)
Antiplatelets	113 (43.6%)
Calcium channel blockers	17 (6.6%)
Diuretics	203 (78.4%)
Digoxin	7 (2.7%)
Nitrate	37 (14.3%)
Statins	147 (56.8%)
Cardiac resynchronisation therapy	21 (8.1%)
Implantable cardioverter defibrillator	23 (8.9%)

**Fig. 1:**
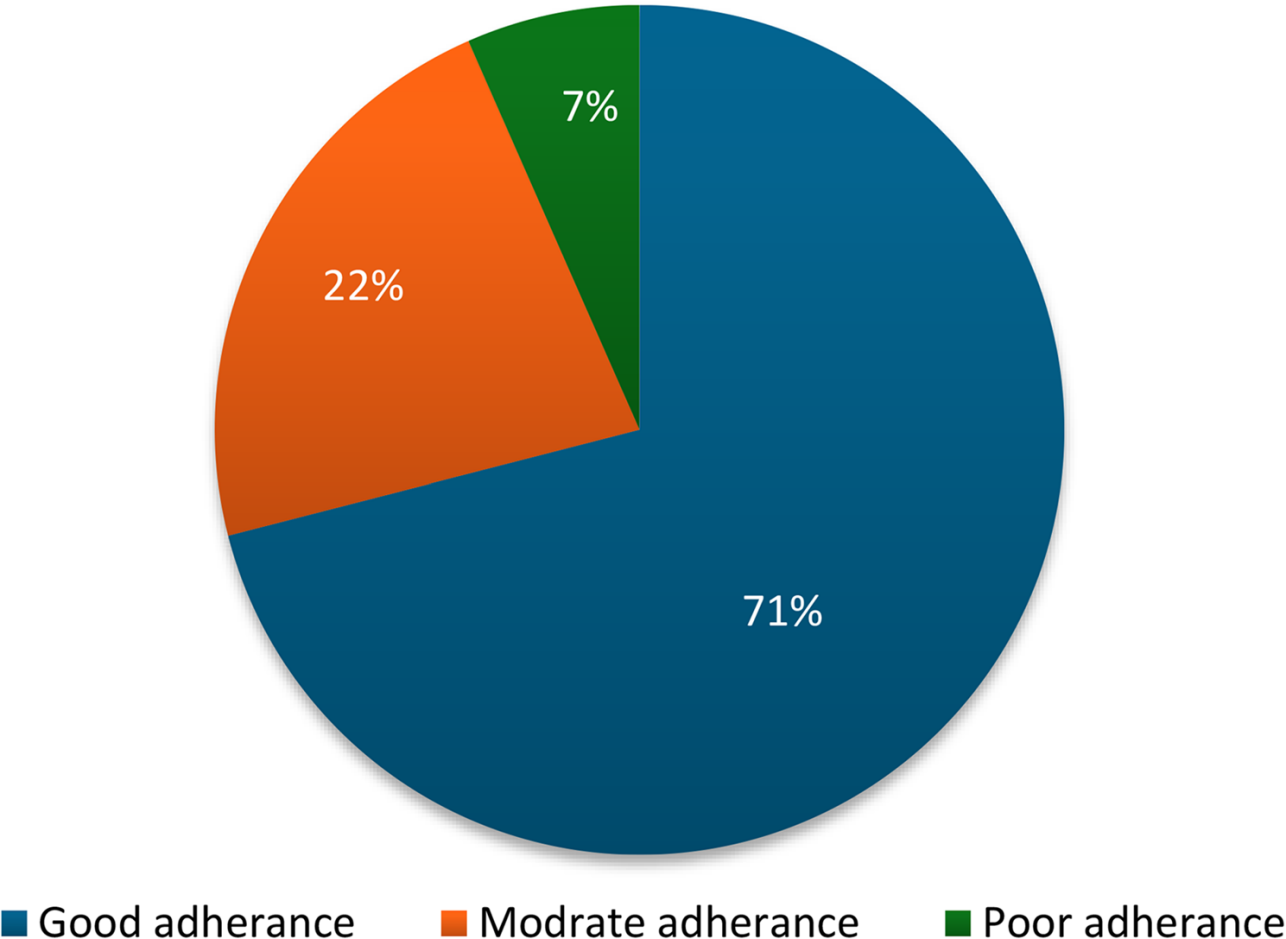
Global guideline adherence score at NHC, Oman. NHC, National Heart Centre; Good adherence, defined as use of all indicated medications in doses ≥50% of the target dose; moderate adherence defined as use of more than half of the medications in doses ≥50% of the target dose; poor adherence defined as and use of less than half the recommended medications and/or in doses <50% of target dose

Based on the Global Guideline Adherence Score (Fig. 1), 71% of patients demonstrated good adherence (i.e., received all indicated GDMT agents at ≥ 50% of target dose), 22% had moderate adherence (more than half of indicated agents at ≥ 50% of target dose), and 7% had poor adherence (less than half of the indicated agents and/or prescribed at < 50% of target dose).

Regarding individual medication classes, target dose attainment was observed in:

- β-blockers (BBs): 78% (203/259),
- ACEI/ARB/ARNI: 63% (165/259),
- MRAs: 96% (248/259),
- Ivabradine: 100% (67/67, among those for whom it was indicated).

**Fig. 2:**
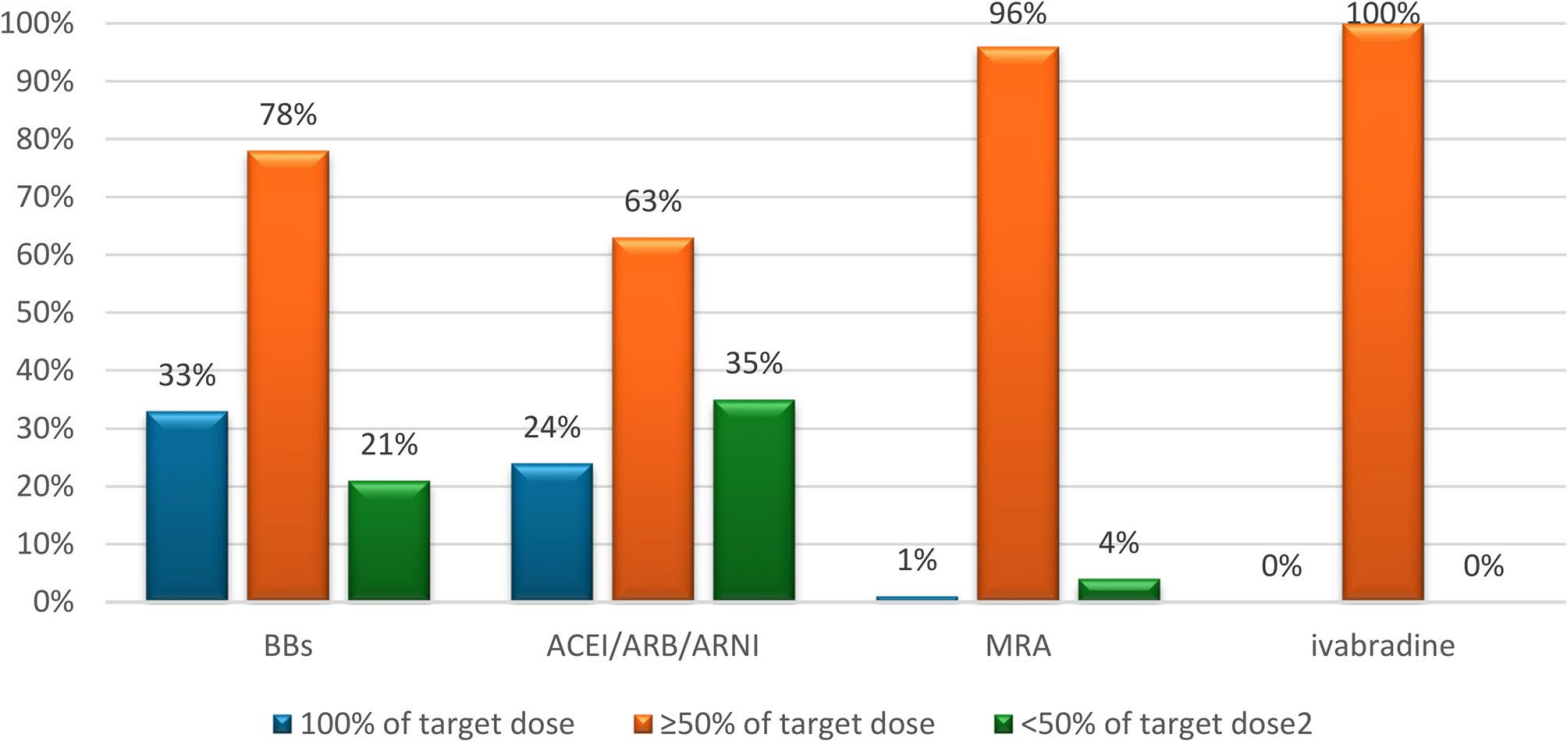
The proportion of GDMT dose attainment. GDMT, guideline-directed medical therapy; BBs, β-blockers; ACEIs, angiotensin-converting enzyme inhibitors; ARBs, angiotensin receptor blockers; ARNIs, angiotensin receptor–neprilysin inhibitors, and MRAs, mineralocorticoid receptor antagonists

Full target dose attainment was reached in:

- 33% (84/259) for BBs,
- 24% (59/259) for ACEI/ARB/ARNI,
- 1% (2/259) for MRAs (Fig. 2).

Univariate analysis identified several factors significantly associated with suboptimal prescribing (< 50% of target dose), as summarized in Table 2:

- Suboptimal β-blocker dosing was significantly associated with HTN (odds ratio [OR]: 2.64; 95% confidence interval [CI]: 1.36–5.13; *P* = 0.004).
- Suboptimal ACEI/ARB/ARNI dosing was associated with DCM (OR: 2.10; 95% CI: 1.18–3.75; *P* = 0.012) and inversely associated with CAD (OR: 0.45; 95% CI: 0.24–0.86; *P* = 0.015).
- Suboptimal MRA dosing was significantly associated with older age (OR: 7.51; 95% CI: 1.93–29.16; *P* = 0.004), CKD (OR: 10.70; 95% CI: 3.05–37.60; *P* = 0.001), and HTN (OR: 3.88; 95% CI: 1.01–14.98; *P* = 0.04).

**Table 2:** Simple logistic regression model results of association between patient-level factors and sub-optimisation (<50%) of BBs, ACEI/ARB/ARNI and MRAs

Variables	BBs		ACEI/ARB/ARNI		MRAs	
	OR (95% CI)	*P*-value	OR (95% CI)	*P*-value	OR (95% CI)	*P*-value
Age per 5 years increase	0.98(0.51–1.88)	0.942	0.56(0.32–0.98)	0.042	7.51(1.93–29.16)	0.004
Female vs. male	1.26(0.65–2.44)	0.496	1.09(0.63–1.90)	0.765	1.96(0.58–6.62)	0.279
EF per 5% increase	0.89(0.49–1.62)	0.707	1.16(0.70–1.94)	0.560	0.53(0.15–1.85)	0.317
NYHA III-IV vs. I-II	1.27(0.40–4.05)	0.685	1.86(0.64–5.36)	0.254	0.72(0.09–6.01)	0.763
ICM	0.96(0.37–2.51)	0.936	0.56(0.25–1.24)	0.152	3.50(0.87–14.08)	0.078
DCM	0.94(0.50–1.76)	0.842	2.10(1.18–3.75)	0.012	0.20(0.03–1.55)	0.122
CKD	1.50(0.55–4.10)	0.431	0.73(0.34–1.58)	0.428	10.70(3.05–37.6)	0.001
HT	2.64(1.36–5.13)	0.004	1.05(0.63–1.76)	0.850	3.88(1.01–14.98)	0.049
DM	1.19(0.63–2.23)	0.596	0.61(0.36–1.04)	0.070	3.42(0.97–12.00.97.00)	0.055
AF	1.24(0.45–3.44)	0.679	1.35(0.57–3.21)	0.500	0.86(0.11–6.94)	0.883
CAD	0.88(0.42–1.87)	0.743	0.45(0.24–0.86)	0.015	1.74(0.44–6.82)	0.428

*Abbreviations*: *BBs* β-blockers, *ACEIs* Angiotensin-converting enzyme inhibitors, *ARBs* Angiotensin receptor blockers, *ARNIs* Angiotensin receptor–neprilysin inhibitors, *MRAs* Mineralocorticoid receptor antagonists, *OR* Odd Ratio, *CI* Confidence Interval, *P*-value Probability value, *EF* Ejection Fraction, *NYHA* New York Heart Association, *ICM* Ischemic Cardiomyopathy, *DCM* Dilated Cardiomyopathy, *CKD* Chronic Kidney Disease, *HT* Hypertension, *DM* Diabetes Mellitus, *AF* Atrial Fibrillation, *CAD* Coronary Artery Disease

## Discussion

In this study, adherence to guideline-recommended heart failure (HF) medications was found to be comparable to that reported in a Polish study by Opolski et al. (2017), which was conducted in a similar tertiary care setting and used the same guideline adherence scoring system [16]. In contrast, Komajda et al. (2016) reported slightly lower adherence rates (67% good, 25% moderate, 8% poor), which may reflect differences in the timing of data collection (2016 vs. 2022) [14]. The Institute of Medicine has highlighted that new clinical evidence can take an average of 17 years to be widely integrated into practice [17, 20], possibly explaining the trend toward improved adherence in more recent studies, including ours.

Notably, our study demonstrated greater success in optimizing GDMT doses (defined as ≥ 50% of target dose for each drug class) compared to earlier reports from Oman. For instance, Hanbali et al. (2020) found that only 56% of patients achieved this threshold for beta-blockers (BBs) and 42% for ACE inhibitors/ARBs [21]. Similarly, Al-Aghbari et al. (2022), even after excluding patients with contraindications, reported optimization in only 61% (BBs) and 44% (ACEs/ARBs) [22]. One key differentiator in our setting was the exclusive management by cardiologists at the National Heart Centre, compared to general practitioners or non-specialist providers in previous studies. This distinction is significant, as studies have shown that non-specialists may be less likely to adhere to HF guidelines or up-titrate GDMT due to factors such as limited awareness of recommendations, focus on symptom relief over long-term outcomes, or fear of side effects [23]. Our findings support the hypothesis that care by HF specialists leads to better adherence and optimization, as also reported in international studies. Nevertheless, it is noteworthy that only 1% of patients in our cohort were prescribed the full target dose of MRAs. This observation suggests that, beyond intolerance, adverse effects, or cost, other factors such as clinical inertia, competing comorbidities, and cautious prescribing practices may have limited titration to maximum doses.

Most cases of suboptimal dosing (< 50% target dose) in our study could be attributed to clinical and physiological factors such as advanced age and comorbid conditions. Ouwerkerk et al. (2017) identified several predictors of under-dosing, including female sex, low BMI, impaired renal function, and lower blood pressure for ACEI/ARBs, while age, low diastolic BP, and signs of pulmonary congestion were associated with lower BB use [9]. Similarly, Greene et al. (2018) found that older patients, those with ventricular arrhythmias, higher ejection fraction, or hyperlipidemia were less likely to be prescribed or up-titrated on GDMT [11].

Age emerged as a consistent barrier to GDMT optimization in our cohort. The concept of the “risk–treatment paradox”, where sicker or older patients receive less aggressive therapy, is well documented [24]. Physicians may overestimate frailty or underestimate benefit in elderly patients, especially since this population is underrepresented in HF clinical trials [25]. Nevertheless, current ESC and ACC/AHA/HFSA guidelines emphasize that age alone should not preclude titration of GDMT, and elderly patients who are not frail or intolerant should still be considered for full therapeutic doses [5, 7, 26].

Collectively, these findings suggest that comorbidities and age-related concerns are major drivers of sub-target dosing in HF care, echoing recent data on guideline adherence gaps [27]. Given that patients with multiple comorbidities are often excluded from trials, clinicians may default to conservative prescribing, inadvertently increasing patients’ risk. Therefore, novel therapeutic strategies with improved safety profiles and careful individualized titration are essential to enhance outcomes in this vulnerable group.

## Study implications and limitations

To our knowledge, this is one of the few studies to evaluate prescribing adherence to GDMT in a specialized ambulatory HF setting in the Gulf region. It highlights important gaps in dose optimization and provides insight into clinical decision-making in a real-world setting. However, several limitations should be noted:

- As a retrospective observational study, causality cannot be inferred.
- The study population was relatively young and predominantly male, which may limit generalizability to older HF patients.
- SGLT2 inhibitors were not included, as they were added to the formulary after the study period.
- We did not assess physician-related or system-level factors that may influence prescribing behaviours, such as awareness of guidelines, patient preferences, or workflow constraints.

Future research should explore these additional factors, including provider-related barriers and institutional influences, to inform multifaceted interventions aimed at improving GDMT uptake and titration.

## Conclusions

In this study, prescribing adherence to guideline-directed medical therapy (GDMT) for patients with heart failure with reduced ejection fraction (HFrEF) in Oman was generally consistent with international findings. However, suboptimal adherence—defined as receiving less than 50% of target GDMT doses—was significantly associated with older age and comorbidities such as hypertension, chronic kidney disease, coronary artery disease, and dilated cardiomyopathy. These results suggest that patient complexity and perceived frailty may influence therapeutic decision-making. As such, adherence to heart failure treatment guidelines should be regularly evaluated within the context of individual patient profiles to support more personalized and effective care strategies.

## Data Availability

Available from the corresponding author upon reasonable request.

## Abbreviations

GDMT: Guidelines-directed medical therapy
HF: heart failure
HFrEF: heart failure reduced ejection fraction
LVEF: left ventricular ejection fraction
HFmrEF: heart failure mildly reduced ejection fraction
HFpEF: heart failure preserved ejection fraction
BBs: β-blockers
ACEIs: angiotensin-converting enzyme inhibitors
ARBs: angiotensin receptor blockers
ARNIs: angiotensin receptor–neprilysin inhibitors
MRAs: mineralocorticoid receptor antagonists
SGLT2Is: sodium-glucose cotransporter-2 inhibitors
PCSK9: proprotein convertase subtilisin/kexin type 9
NHC: National Heart Centre
ESC: European Society of Cardiology
HT: hypertension
DM: diabetes mellitus
DCM: dilated cardiomyopathy
CAD: coronary artery disease
CKD: chronic kidney disease
EF: ejection fraction
